# Soil Microbial Community Structure and Diversity around the Aging Oil Sludge in Yellow River Delta as Determined by High-Throughput Sequencing

**DOI:** 10.1155/2018/7861805

**Published:** 2018-08-30

**Authors:** Shaoping Kuang, Yaqing Su, Huihui Wang, Wenjuan Yu, Qiaolin Lang, Ravichandra Matangi

**Affiliations:** ^1^College of Environment and Safety Engineering, Qingdao University of Science and Technology, Qingdao, Shandong Province 266042, China; ^2^Advanced Analytical Laboratory, DST-PURSE Programme, Andhra University, Visakhapatnam 53003, India

## Abstract

Microorganisms are sensitive indicators of edaphic environmental variation. The Illumina MiSeq sequencing technology was used to analyze soil bacterial community diversity around an aging oil sludge in the Yellow River Delta. The alpha diversity index of soil bacterial community results (Ace, Chao, Shannon, and Simpson) determined that bacterial community diversity sampling within the scope of a 20 cm radius from the center of an aging oil sludge spot showed the most abundant diversity. The level of diversity distributed symmetrically with radial direction from the center of the aging oil sludge spot. Over the distance of 100 m from the center, bacterial community diversity tends to be monotonous, with small differences especially in the horizontal direction underground. The alpha-diversity indicators also showed that the bacterial diversity of samples were close under the aging oil sludge. In addition, the aging oil sludge inhibited the growth of bacteria compared with the referenced unpolluted soil sample and also increased the diversities of soil bacteria. At the phylum level, the *Proteobacteria*, *Chloroflexi*, and *Actinobacteria* existing in the aging oil sludge-contaminated wetland soil constituted a larger proportion of the community, while the proportion of *Firmicute* was relatively less. On the contrary, *Firmicute* showed the highest content of 63.8% in the referenced soil. Under the genus level and family level, the corresponding strains that resisted the aging oil sludge were selected. According to the bacterial diversity analysis, the basic structure of the bacterial community which could be used for remediation of aging oil sludge-contaminated soil was also developed.

## 1. Introduction

Microorganisms play an important role in the soil environment. The variation of the microbial populations often indicates the change of the soil environment. Environmental pollution may cause a transformation in microbial community composition and activity [1, 2]. Moreover, there is a dependent relation between microbial diversity and soil contamination [1, 3]. The stability of microbial diversity represents the status of a microbial community, which could be used to predict the transformation trend of the environmental quality and soil nutrient conditions. Thus, it is considered to be one of the most sensitive biological indicators. Soil microorganisms occupy an important position in the cycle of the biological geochemical system. Simultaneously, it plays an important role in soil self-purification, toxic compound transition, and transformation of the soil environment. Soil microorganisms are far more sensitive to contaminants than soil animals and plants [4], which is an indicator of the changes in the physical and chemical properties of the soil and environmental quality.

With the increasing demand of petroleum, oil production is increasing sharply, leading to incidents of serious soil pollution. The Shengli Oil Field, located in the Yellow River Delta, is the second largest oilfield in China. However, there is a National Nature Reserve in the Yellow River Delta with hundreds of animals and plants that need to be protected. It also has wetlands that cover an area of over 4500 km^2^. Obviously, the oil production process has a negative effect on the protection of endangered organisms and wetlands. Especially, the aging oil sludge (long-term untreated oil spots scattering around oil wells), with the potential risk of longtime and rock-ribbed pollution to the surroundings, presents a thorny problem of treatment and disposal. Meanwhile, a lot of toxic substances are included in the oil sludge. For example, the soil around a crude oil storage site in the Jianghan Oil Field of China was severely contaminated with polycyclic aromatic hydrocarbons (PAHs). The soil around the oil sludge or in the oil exploration area was similarly contaminated with PAHs [5–7].

However, much attention has been paid to the stacking oil sludge in the open air [8–10]. The scattered oil sludge in oil-producing regions, especially around the oil wells, were ignored for years, forming a lot of aging oil sludge-contaminated sites on the soil. Until now, the quantities of the most deleterious components in the aging oil sludge [11], such as total petroleum hydrocarbons (TPHs) and heavy metals, as well as bacterial diversity of the long-time oil-polluted soil, are still unknown and rarely referred to and reported globally [12].

Currently, high-throughput sequencing is recognized as a powerful method to analyze a bacterial community. Its specific primers are also known as accurately providing the diversity of bacterial groups at a fine scale [13, 14]. The MiSeq sequencing system adopted the mature TruSeq with synthetic sequencing technology simultaneously, which integrated amplification, sequencing, and data analysis in one machine. Illumina technology was commonly used as the foundation with the method of the reversible termination reagent, which simultaneously detects millions of pieces at a large-scale. To add each dNTP, the terminator is imaged with fluorescent tags and subsequently cut. Because four kinds of reversible termination were combined with dNTPs, natural competition with minimum deviation detects the base sequence directly by virtue of each cycle of a fluorescence signal. Now, the high-throughput sequencing method is widely used in the process of microbial diversity analysis, since it has the characteristics of no culture, high sensitivity, and low detection limit [15, 16].

It was reported that the types and quantities of microorganisms were closely associated with the contents of soil moisture, organic carbon, soil clay, and soil microorganisms [17]. Researchers analyzed the characteristics of microbial diversity in the Yellow River Delta wetland. The results showed that the number of microorganisms and the diversity of cultured microorganisms decreased with the increase of inorganic salt [18, 19]. Moreover, the geochemical parameters such as pH, Eh, As, sulfate, and water temperature also had significant effects on the indigenous microbial community [20].

This work mainly researched on the transformation of the bacterial community structure caused by the aging oil sludge using high-throughput sequencing technology [2, 9, 10, 21]. The 16S rRNA gene analysis and taxonomical analysis were performed with a clone library. The results and related findings would aid in a thorough understanding of the microorganism structure in aging oil sludge-contaminated soil and thus provide a new point of view to soil bioremediation.

## 2. Materials and Methods

### 2.1. Experimental Setup

The soil samples in this experiment were collected from the Shengli Oil Field in Shandong Province, China. Meanwhile, the test soil was obtained from three separate sites across the oil field. Three points in various directions around the aging oil sludge were selected, 0 and 20 cm below the earth of the aging oil sludge, and over 100 m in horizontal direction from the aging oil sludge; the latter was used as the corresponding reference soil.

#### 2.1.1. Sampling and Processing

1 kg each of the three soil samples was collected, removing the stones and plant debris with the tube labeled for sampling time, sampling site, sampling code, and the surrounding geographical environment outside. Soil samples were preserved at a temperature of 4°C after being transported to the laboratory. A four-point method was used to remove excess soil and the soil finally remained as representative samples. Soil samples were dried naturally and were passed through 100-mesh sieves to determine their physical and chemical properties.

#### 2.1.2. Microbial Diversity Experiment

A sterilized shovel was used to collect soil samples around the aging oil sludge while removing the stones and debris. Finally, 50 g of soil was collected as a representative sample. Meanwhile, there was a need to change to a new sterilized shovel for each soil sample to prevent cross-contamination. The representative samples were preserved in sterile tubes and marked for sampling time, sampling site, sampling code, and surrounding geographical environment outside the tubes. Soil samples were transported with carbon dioxide, and then stored with liquid nitrogen under the temperature of −80°C immediately.

### 2.2. Analytical Methods

#### 2.2.1. Determination of Soil Physical and Chemical Properties

Moisture content was determined by weighing samples before and after oven drying at 105°C for 24 h. The pH was measured with a pH meter (Mettle-Toledo Instruments, Shanghai, China). The determination of the soil organic carbon was referred to GB 9834-1988. Heavy metals, including copper, zinc, and chromium, were measured by an atomic absorption spectrophotometer (GFA-7000, Japan). The lights of the microwave digestion method were used and the acid system was nitric acid, hydrofluoric acid, and hydrogen peroxide. Experimental steps were as follows: first, 0.25 g of soil sample was accurately put in a microwave digestion jar. Then, 5 ml of nitric acid, 2 ml of hydrofluoric acid, and 1 ml of hydrogen peroxide were added successively. Then, the appropriate temperature and time were set to cool down the soil samples. In addition, the digestion liquids were transferred to a 50 ml beaker for digesting. After a period of cooling and dissolving the residue, the solution was transferred to a 50 ml volumetric flask with 5 ml of 5% La (NO_3_)_3_ (Cu/Zn) or 5 ml of 10% NH_4_Cl (Cr). Besides, the determination of total petroleum hydrocarbons (TPH) was referred to HJ/T 350-2007, China.

#### 2.2.2. DNA Extraction, 16S rRNA Gene Amplification, and High Throughput Sequencing

Soil microbial metagenomic DNA was isolated with a Soil DNA Kit (Omega Bio-tek, Norcross, GA, US) according to the manufacturer's instruction. The DNA extracts were stored at −20°C for the following PCR amplification. The universal 16S rRNA gene primers were the 515F (5′-GTGCCAGCMGCCGCGG-3′) and 806R (5′-GGACTACHVGGGTWTCTAAT-3′). The barcode and adapter were incorporated between the adapter and the forward primers. The PCR used was the TransStart FastPfu DNA Polymerase, a 20 *μ*L reaction system. The PCR mixture contained 10 ng of DNA template, 2 *μ*L of 2.5 mM dNTPs, 0.8 *μ*L of both primers, 0.4 *μ*L of FastPfu Polymerase, 4 *μ*L of 5x FastPfu Buffer, and sterile double-distilled H_2_O in a total volume of 20 *μ*L of PCR amplification which was performed in a ABI GeneAmp 9700 (USA). The PCR process consisted of an initial 5 min denaturation at 95°C, followed by 27 cycles of denaturing at 95°C for 30 s, and annealing at 55°C for 30 s, with an extension at 72°C for 45 s. The same sample was mixed with the PCR product with 2% agarose gel electrophoresis detection. By virtue of the AxyPrep DNA gel extraction kit (Axygen Biosciences, Union City, CA, US) PCR products were cut, eluted with Tris-HCl, and subjected to 2% agarose electrophoresis detection at the same time. The QuantiFluor™-ST (Promega, US) blue fluorescence quantitative system for PCR products was used for detection. The next step was building the MiSeq library. We chose the TruSeq™ DNA Sample Prep Kit and cBot TruSeq PE Cluster Kit v3-cBot-HS. Specific steps were as follows: the P7 and P5 joints were connected; the magnetic bead filter was used to remove the irregular fragments; PCR amplification was used to enrich the library template; and alkali degeneration was used to produce single-stranded DNA fragments. The cBot Truseq PE Cluster Kit v3-cBot-HS was the cluster-generation kit for bridge amplification.

The treated samples in the MiSeq PE300 platform were sequenced for about 65 hours. The end DNA fragments complemented with the primer base and were fixed on a chip, while the other end complemented randomly with the other primers, and was then fixed and formed a “bridge.” PCR amplification and DNA clusters were produced at the same time. Subsequently, DNA linearization was performed while producing a single DNA strand. The modified DNA polymerase and four kinds of fluorescence-labeled dNTP were added while each cycle used only a synthetic base. The polymerization nucleotide species in each template was obtained by scanning the plate reaction surface with a laser. The chemical cutting of the “fluorescent groups” and “end groups” was carried out, and the viscosity ending of 3′ was restored. Meanwhile, the second nucleotide was aggregated and the results of fluorescent signal in each round were calculated and collected, while the template DNA sequence was obtained.

#### 2.2.3. Sequencing Data Processing

The overlap relationship was used to obtain the PE reads while carrying out quality control and filtering the quality of the sequence at the same time. The OTU taxonomists' analysis, cluster analysis, and diversity index analysis were carried out after distinguishing the samples. Meanwhile, we conducted various diversity index analyses. The detection of sequencing depth was also conducted on the basis of OTU clustering analysis. The community structure analysis in each classification level was conducted by the taxonomy information. On the basis of the analysis above, the study was carried out on a serial analysis of community structure, system development, and visualization.

According to the similarity levels, all the sequences were taken using OTU division. Meanwhile, by dint of the OTU biological information, under a 97% similarity level, the statistical analysis was carried out. Analysis steps were as follows: extracting the nonrepeated sequence from an optimized sequence to reduce the redundant computation in the analysis course (http://drive5.com/usearch/manual/dereplication.html); removing the nonrepeated single sequence (http://drive5.com/usearch/manual/singletons.html); and conducting OTU cluster on nonrepeated sequences and removing the chimera in the process of clustering while obtaining a representative sequence of OTU under a similarity of 97%. All the optimized sequences were mapped to the representative OTU sequence, and the sequences which own the similarity level by more than 97% were selected while generating the OTU form.

To acquire the information of the corresponding species classification for each OTU, the RDP classifier Bayesian algorithm analyzed representative sequences of OTU under the 97% similarity level and calculated the community composition in each classification level. The databases were as follows: Silva (release 119 http://www.arb-silva.de) and RDP (release with the 11.1 http://rdp.cme.msu.edu/). The rarefaction curve was obtained from the sequencing depth of the sample. Rarefaction analysis was conducted with 97% similarity OTU, using mothur and R language tools to make a graph. Bacterial diversity indices were as follows: Chao—the Chao1 estimator (http://www.mothur.org/wiki/Chao); Ace—the ACE estimator (http://www.mothur.org/wiki/Ace); Shannon—the Shannon index (http://www.mothur.org/wiki/Shannon); Simpson—the Simpson index (http://www.mothur.org/wiki/Simpson); and the index of the sequencing depth coverage—the Good's coverage (http://www.mothur.org/wiki/Coverage). According to the analysis of beta diversity for a hierarchical clustering distance matrix, the group used the method of UPGMA (unweighted pair group method with arithmetic mean) to construct a tree structure.

### 2.3. Statistical Analysis

Statistical analysis was carried out with SPSS 19.0 software and Origin 8.0.

## 3. Results

### 3.1. Physical and Chemical Characteristics

The physical and chemical properties of the soil were summarized in Table 1. Results demonstrated that the contaminated soils were slightly alkaline and generally had a higher pH value than the corresponding reference soils. According to the soil and environmental quality standard, the heavy metal contents including copper, zinc, and chromium in aging oil sludge-contaminated soils were not fit for planting. Meanwhile, the heavy metal content could cause serious damage and pollution to the soil. Similarly, the contaminated soil showed an elevated level of organic matter (organic carbon) with a range of 0.05%–0.41%. Compared with the reference value, the content of total organic carbon was moderate. Moreover, the results of TPH demonstrated that petroleum hydrocarbons were detected in the soil which was closest to the aging oil sludge.

### 3.2. MiSeq-Pyrosequencing Results and Microbial Structures

16S rRNA sequencing had provided a detailed view on the composition. The analysis of the single sample diversity (alpha diversity) could reflect the richness and the diversity of the microbial community. The study aimed at analyzing the bacterial diversity of the soil contaminated with the aging oil sludge. The soil sample obtained from 20 cm below the earth of the aging oil sludge was marked as number 1. Sample number 2 was fetched below the aging oil sludge directly. Sample number 3 was the plain soil more than 100 m distant from the aging oil sludge. Meanwhile, number 3 was used as the corresponding reference soil. The sequence information and microbial diversity index of the samples are listed in Table 2. OTU numbers of the three samples were 1452, 1280, and 747, respectively. Among the entire diversity index, Ace estimated the OTU number in the community, while Chao estimated the OTU number in the soil sample. In other words, Ace and Chao indicated the community richness. In addition, the value of Shannon estimated the microbial diversity, positively associated with microbial diversity. On the contrary, Simpson was used to estimate microbial diversity, the value of which was negatively correlated with microbial diversity. Meanwhile, coverage was the probability of the measured sample sequence.

Index results showed that the numbers of Ace, Chao, Shannon, and Simpson had no significant differences between sample numbers 1 and 2. It was clear that the Ace, Chao, and Shannon were higher in the aging oil sludge-contaminated soil than in the corresponding reference soil, while the Simpson was lower than that of the corresponding reference soil. It was concluded that soil samples under the aging oil sludge showed the highest content on bacterial diversity. Similarly, bacterial diversity was lower in soil which was directly under the aging oil sludge. The numerical value also showed that sample number 3, obtained at a location furthest away from the aging oil sludge, showed the lowest bacterial diversity. It was obvious that samples from the furthest distance to the aging oil sludge presented the lowest microbial diversity. Moreover, the alpha diversity also indicated that the aging oil sludge inhibited microbial diversity to a certain extent, as we found out from the soil 0 cm under the aging oil sludge, while we could also see that the aging oil sludge might produce an increase of bacterial diversity from the soil 20 cm under the aging oil sludge. This explains why different distances from the aging oil sludge leads to soil microbial populations with different microbial diversity.

**Figure 1:**
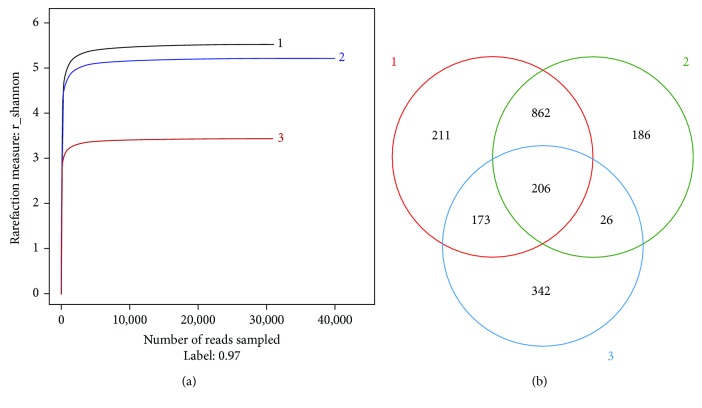
Rarefaction curves and OTU Venn analysis in different samples.

A rarefaction curve was built with the numbers of selected individuals from a sample with certain quantity and species numbers counted from the individuals represented. The rarefaction curve was used to indicate whether the sequencing quantity was enough. It was commonly used to compare the sequencing data volume of species richness in different samples and to indicate whether the quantity of sequencing data were reasonable. A smooth curve meant reasonable sequencing data, and much more data only produced a little new OTU. As illustrated in Figure 1(a), the result in the study demonstrated that all the aging oil sludge-contaminated soil had a similar pattern, which was different from the corresponding reference soil. Within a certain range, the curves with a sharp-rise trend indicate that a lot of species had been found in the community. A flattened curve meant that the species would not significantly increase with the increase of sample size in the environment. Meanwhile, the result also showed that the sequencing number was easier to reach in the corresponding reference soil. The study similarly illustrated that the corresponding reference soil had the lowest richness, and a little sequencing number could also perfectly reflect bacterial diversity in the samples.

Venn analysis was used for counting the number of common and unique OTU in different samples with the similar level of 97%.  Figure 1(b) shows that the OTU numbers were 1452, 1280, and 747 in the three samples. The proportion of unique OTU was 10.5% in the number 1 soil and 9.3% in the number 2 soil. Moreover, the proportion of unique OTU was 17% in the corresponding reference soil. In addition, the common OTU in aging oil sludge-contaminated soil was 53.2%, while the proportion of the common OTU was 10.3% in three samples.

### 3.3. Taxonomic Complexity of Bacterial Community

**Figure 2:**
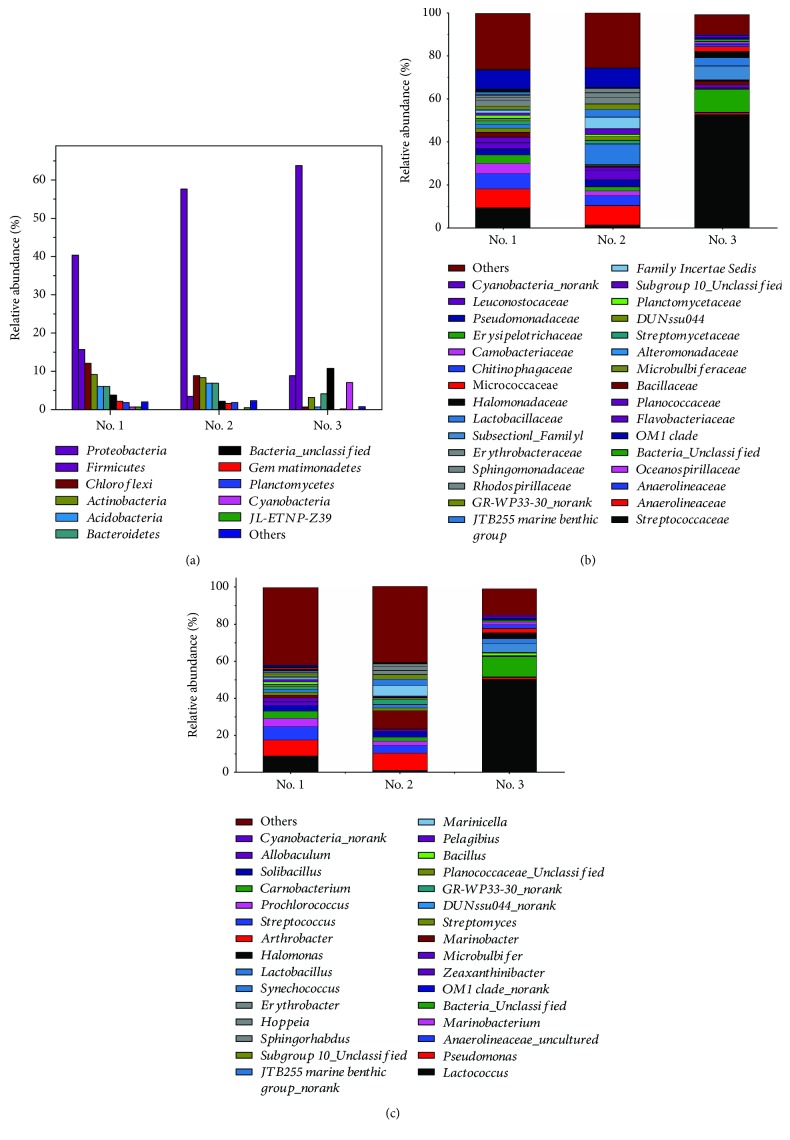
The bacterial histogram of different samples.

**Figure 3:**
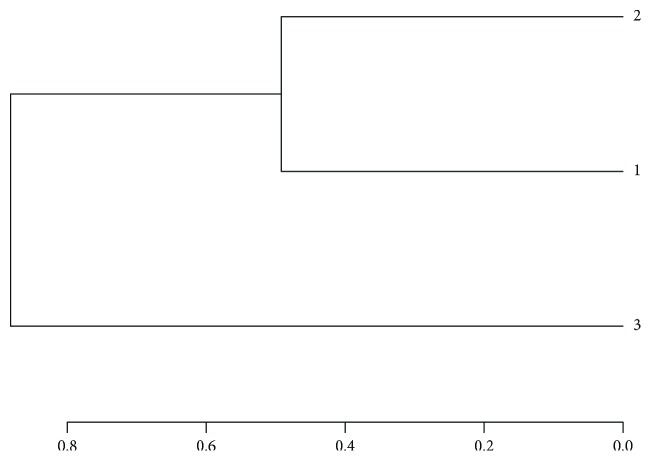
Multiple sample similarity tree.

The richness of the bacterial community in different levels was illustrated in Figure 2. As shown in Figure 2(a), the dominant phyla in aging oil sludge-contaminated soil were mainly *Proteobacteria*, *Chloroflexi*, *Actinobacteria*, *Acidobacteria*, *Bacteroidetes*, and *Firmicute*, with a higher content in samples under the aging oil sludge. *Proteobacteria*, *Firmicute*, *Actinobacteria*, *Cyanobacteria*, and *Bacteroidetes* were the main bacteria in the corresponding reference soil. *Proteobacteria* was the highest phylum in the aging oil sludge-contaminated soil, with the relative content of 40.30% and 57.60% in aging oil sludge-contaminated soil, respectively, while it was 8.89% in the corresponding reference soil. Moreover, the highest phylum in the corresponding reference soil was *Firmicute*, with a relative content of 63.81%; however, it was reduced sharply in the aging oil sludge-contaminated soil. In addition, *Chloroflexi* was 12.05% and 8.70% in soil sample number 1 and number 2, respectively, while the content was 0.56% in the corresponding reference soil. The change trend of *Acidobacteria* is the same with *Chloroflexi* in the three samples. In general, these results reflected the differences and relationships of phylum diversity in the three samples. The richness of the bacterial community at the family level was illustrated in Figure 2(b). From the picture, we could see that *Pseudomonadace*, *Anaerolineaceae*, *Oceanospirillaceae*, *Flavobacteriaceae*, and *Pseudomonadaceae* are the main families in the aging oil sludge-contaminated soil. The contents of *Streptococcacea* were 9.16% and 1.34% respectively in soil 20 cm and 0 cm below the earth of the aging oil sludge, which indicated that the aging oil sludge might have different effects on bacterial diversity. *Streptococcaceae*, *SubsectionI_Family I*, *Lactobacillaceae*, and *Halomonadaceae* were the dominant species in the corresponding reference soil. *Oceanospirillaceae* showed a higher content in the aging oil sludge-contaminated soil but there was no *Oceanospirillaceae* in the corresponding reference soil. Similarly, *Streptococcaceae* was the highest family in the reference soil and the percentage of *Streptococcaceae* was 52.35%. The level of genus bacterial diversity (Figure 2(c)) reflected that *Pseudomonas*, *Anaerolineaceae_uncultured*, *Marinobacterium*, and *Sphingorhabdus zeaxanthinibacter* were the dominant genera in the aging oil sludge-contaminated soil. It was obvious that *Lactococcus* and *Synechococcus* are the main genera in the reference soil. The content of *Lactococcus* was 50.32% in the corresponding reference soil and 8.80% and 1.29% in soil 20 cm and 0 cm below the earth of the aging oil sludge. The results demonstrated that the percentage of *Zeaxanthinibacter* was 4.56% and 2.28% in the aging oil sludge-contaminated soil, but there was none in the corresponding reference soil. In addition, the clustering analysis method was used in the study and the branch length represented the distance in different samples. The short range of branches meant that the species composition of the sample was much more similar. Similarly, the branch structure was used to describe and compare similarities and differences between multiple samples. As illustrated in Figure 3, the aging oil sludge-contaminated soil was classified together and the corresponding reference soil was classified separately.

**Figure 4:**
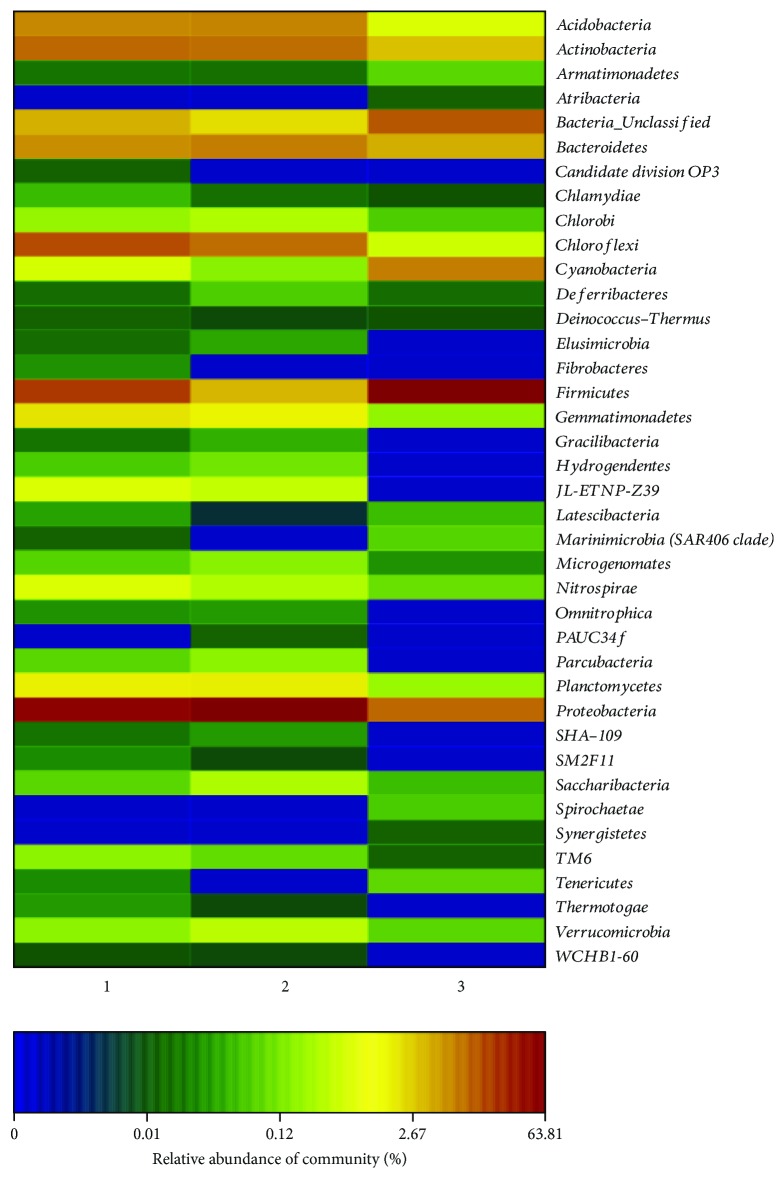
Bacterial community heat map analysis.

High-richness and low-richness species were partitioned and gathered together with a heat map. It reflected the similarities as well as the differences of the sample community composition at the phylum classification level in virtue of the color gradient and similar degree.  Figure 4 shows the bacterial community analyzed under the level of phylum. The number of phyla which hardly existed, respectively, was 4, 7, and 13 among all the soil samples, respectively. The three samples, having no phylum, were 0. Some communities, such as *Omnitrophica*, *Marinimicrobia*, and *Bacteroidetes*, were few in all the samples. After our analysis and statistics, *Proteobacteria*, *Firmicutes*, *Chloroflexi*, *Actinobacteria*, *Acidobacteria*, *Bacteroidetes*, *Gemmatimonadetes*, and *Planctomycetes* were high (>1%) in 20 cm soil contaminated with the aging oil sludge. Furthermore, the contents of *Proteobacteria*, *Chloroflexi*, *Actinobacteria*, *Acidobacteria*, *Bacteroidetes*, *Firmicutes*, *Gemmatimonadetes*, and *Planctomycetes* were all over 1% in the 0 cm soil contaminated with the aging oil sludge. *Proteobacteria*, *Firmicutes*, *Actinobacteria*, *Bacteroidetes*, and *Cyanobacteria* were more than 1% in the corresponding reference soil. The content of *Chloroflexi*, *Acidobacteria*, *Gemmatimonadetes*, and *Planctomycetes* in the reference soil was lower compared with the aging oil sludge-contaminated soil. In addition, the content of *Proteobacteria* in the aging oil sludge-contaminated soil was high while the content reduced sharply in the corresponding reference soil. Inversely, the content of *Firmicute* plummeted in the aging oil sludge-contaminated soil, but it was the highest phylum in the corresponding reference soil.

## 4. Discussion

The community structure of soil microorganisms was always related to the soil physical and chemical properties. He et al. showed that soil properties could significantly change the richness, composition, and structure of microbial species, which might improve and modify ecosystem function [22, 23]. The results of physical and chemical properties showed that the soil was alkaline. pH must be taken into account for its effect in the growth of bacteria. A decreasing pH and increased metal contamination showed a negative effect on bacterial growth [24]. It may be for this reason that sample 1 has the largest bacterial diversity. Moisture could affect microbial activity, transcription, and composition [25]. The moisture contents in sample 1 (26.6%) and sample 2 (21.1%) were generally higher compared with sample 3 (14.0%) which was inconsistent with previous studies [9, 10, 26]. It is probably the hydrophobic crusts formed by the heavy oil components in AOS which limited the evaporation of water and the water/air exchange of soil [27]. The results of the soil's physical and chemical properties indicated that the aging oil sludge had little effect on the receiving soils. In general, the high pH indicated that the aging oil sludge-contaminated soil was not suitable for planting; the soil should be cultivated with alkali plants. It could not only improve soil pH, but could also maintain soil moisture. In addition, this method could also improve soil physical and chemical properties and make the soil suited for farming.

Bacterial richness and diversity were usually considered as biological indicators of the origin for soil aggregates [28]. MiSeq sequencing revealed significant differences in the microbial taxonomic composition between the contaminated soil and corresponding reference soil. In total, 9 phyla were identified and *Frimicute* and *Proteobacteria* were widely recognized as the predominant phyla in the soil. In the background soil sample, *Frimicute* was the dominant phylum. While in the contaminated soil samples, *Frimicute* decreased sharply and *Proteobacteria* and *Chloroflexi* became the dominant phyla. In other words, *Firmicute* was sensitive to the aging oil sludge. Without the influence of the aging oil sludge, the richness of *Firmicute* decreased dramatically. The aging oil sludge had no adverse effects on *Proteobacteria* and *Chloroflexi*, which increased the relative richness of the two phyla. Namely, the two phyla might be the functional bacterium for oil degradation.

Further analysis was made to present the richness and diversity of the bacterial community at the family level. *Streptococcaceae* was the dominant bacteria in the corresponding reference soil and maintained a little content in the aging oil sludge-contaminated soil. Besides, *Pseudomonadaceae* showed a high content in the aging oil sludge-contaminated soil, while it decreased dramatically in the corresponding reference soil. The number was 20 in the aging oil sludge-contaminated soil. The community richness of *Streptococcaceae* decreased significantly in the three soils. However, the richness of *Pseudomonas* and *Alteromonadaceae* increased, which meant that *Streptococcaceae* was sensitive to the aging oil sludge. Moreover, the richness of *Streptococcaceae* reduced rapidly in the aging oil sludge-contaminated soil, while the relative abundance of *Pseudomonas* and *Alteromonadaceae* increased. Analysis results indicated that *Pseudomonas* and *Alteromonadaceae* have better resistance in the aging oil sludge. The shrinking of *Streptococcaceae* provided greater living space to *Pseudomonas* and *Alteromonadaceae*. Moreover, these two strains of microorganisms existed to degrade the contaminant in the aging oil sludge. Previous studies repeatedly demonstrated that *Acinetobacter* and *Pseudomonas* could degrade toxic organic compounds [29–31], which was of great significance to the microbial remediation of the aging oil sludge.

Under the level of genus, *Lactococcus* was the dominant bacteria in the corresponding reference soil and *Pseudomonas* showed a high content in the aging oil sludge-contaminated soil. The study showed that bacterial diversity was richer in the aging oil sludge-contaminated soil, from which we could choose bacteria to resist the aging oil sludge. Bacteria which resisted the aging oil sludge could lay a foundation for bioremediation in the aging oil sludge-contaminated soil.

Soil used in the study was obtained from the Shengli Oil Field in China, which had a long history of contamination with petroleum hydrocarbons [32]. High-throughput sequencing had enabled in-depth exploration of microbial diversity in the environment. In addition, the Ace and Chao were the indicators which indicated microbial richness, while the Shannon-Weaver and Simpson indices reflected microbial diversity [33]. The bacterial diversity index showed that the aging oil sludge significantly affected the microbial diversity. The contrast analysis of all the soil samples demonstrated that microbial species were richer in the aging oil sludge-contaminated soil. As a result, the soil at a 20 cm vertical distance under the aging oil sludge showed higher community diversity than the surface of the earth and the corresponding reference soil. Different soils contaminated with different degrees of the aging oil sludge indicated that the aging oil sludge not only could promote bacteria microbial diversity but could inhibit bacteria microbial diversity. The study also found that the aging oil sludge could significantly influence bacterial diversity among different pollution degrees. Moreover, we also selected the dominant microorganisms in soil contaminated with the aging oil sludge. In a subsequent study, the researchers planned to use the microorganisms which resisted the aging oil sludge to restore contaminated soil and especially decrease the content of petroleum hydrocarbons.

## 5. Conclusions

The study investigated soil bacterial diversity around an aging oil sludge and analyzed the community structure and richness of bacteria. The research results showed that the aging oil sludge could significantly affect the growth of soil bacteria and inhibit the growth of bacteria. Under different pollution degrees of the aging oil sludge, the results showed different bacterial diversity. At the phylum level, the *Proteobacteria*, *Chloroflexi*, and *Actinobacteria* existing in the aging oil sludge-contaminated wetland soil constituted a larger proportion of the community, while the proportion of *Firmicute* was relatively less. On the contrary, *Firmicute* showed the highest content of 63.8% in the referenced soil. Under the genus and family levels, the corresponding strains that resisted the aging oil sludge were selected. According to the bacterial diversity analysis, the resistance bacteria laid a foundation for the subsequent soil bioremediation.

## Figures and Tables

**Table 1:** The soil physical and chemical properties.

Sample ID	pH	Moi (%)	TOC (%)	Cu (mg/kg)	Zn (mg/kg)	Cr (mg/kg)	TPH (mg/kg)
Number 1	8.55	26.6	0.05	47.93	93.81	111.46	15.2
Number 2	8.44	21.1	0.41	76.60	131.63	74.55	<5
Number 3	8.11	14.0	0.22	12.20	15.68	34.07	<5

**Table 2:** Different bacterial diversity indices in different samples.

Sample ID	Ace	Chao	Shannon	Simpson	Coverage
Number 1	1577	1563	5.52	0.0185	0.993
Number 2	1348	1349	5.21	0.0237	0.996
Number 3	760	757	3.43	0.1780	0.999

## Data Availability

The data used to support the findings of this study are available from the corresponding author upon request.
